# Cnidofest 2022: hot topics in cnidarian research

**DOI:** 10.1186/s13227-023-00217-9

**Published:** 2023-08-24

**Authors:** James M. Gahan, Paulyn Cartwright, Matthew L. Nicotra, Christine E. Schnitzler, Patrick R. H. Steinmetz, Celina E. Juliano

**Affiliations:** 1 Department of Biochemistry, University of Oxford, Oxford, OX1 3QU UK; 2 Michael Sars Centre, University of Bergen, Thormøhlensgt. 55, 5008 Bergen, Norway; 3 Department of Ecology and Evolutionary Biology, University of Kansas, Lawrence, KS 66045 USA; 4 Thomas E. Starzl Transplantation Institute, University of Pittsburgh, Pittsburgh, PA 15261 USA; 5 Center for Evolutionary Biology and Medicine, University of Pittsburgh, Pittsburgh, PA 15261 USA; 6 Department of Immunology, University of Pittsburgh, Pittsburgh, PA 15213 USA; 7 Whitney Laboratory for Marine Bioscience and Department of Biology, University of Florida, St. Augustine, FL 32080 USA; 8 Department of Molecular and Cellular Biology, University of California, Davis, CA 95616 USA

**Keywords:** Cnidofest, Cnidarians, Conference, *Hydra*, *Nematostella*, *Hydractinia*, *Clytia*

## Abstract

The second annual Cnidarian Model Systems Meeting, aka “Cnidofest”, took place in Davis, California from 7 to 10th of September, 2022. The meeting brought together scientists using cnidarians to study molecular and cellular biology, development and regeneration, evo-devo, neurobiology, symbiosis, physiology, and comparative genomics. The diversity of topics and species represented in presentations highlighted the importance and versatility of cnidarians in addressing a wide variety of biological questions. In keeping with the spirit of the first meeting (and its predecessor, Hydroidfest), almost 75% of oral presentations were given by early career researchers (i.e., graduate students and postdocs). In this review, we present research highlights from the meeting.

## Introduction

**Fig. 1:**
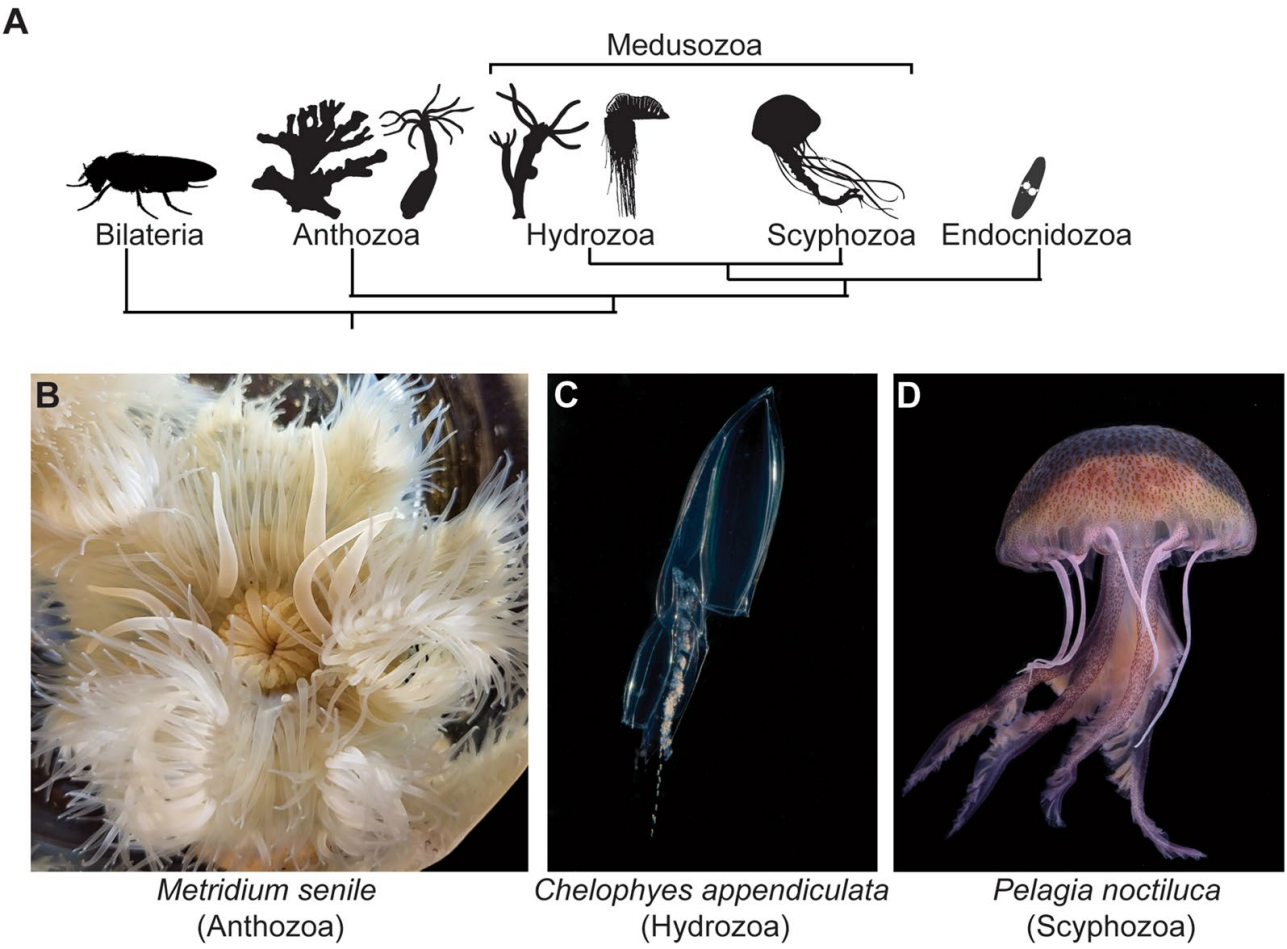
Cnidarian phylogeny. **A** A simplified phylogenetic tree showing the position of cnidarians as the sister group to the Bilateria and the relationship between major groups of cnidarians presented at the meeting [45]. The Cnidaria can be spilt into three groups; Anthozoa (Sea anemones, corals etc.); Medusozoa (hydroids, siphonophores, true jellyfish); Endocnidozoa (parasitic Myxozoa and *Polypodium*). **B**–**D** Examples of organisms presented at the meeting: the sea anemone *Metridium senile* (Anthozoa) (**B**), a polygastric colony of the siphonophore *Chelophyes appendiculata* (Hydrozoa) (**C**) and an adult medusa of the jellyfish *Pelagia noctiluca* (Scyphozoa). Image credits: Yareli Alvarez (**A**), Maciej Mańko (**B**) and Alexandre Jan (**D**). The silhouettes in A were obtained from phylopic (https://www.phylopic.org/) and all the images are available under a CC0 1.0 license except the siphonophore which was produced by Noah Schlottman (photo by Casey Dunn) and is available under a CC BY-SA 3.0 license (https://creativecommons.org/licenses/by-sa/3.0/)

The phylum Cnidaria is a diverse group of aquatic animals (mostly marine) which includes hydroids, jellyfish, sea anemones, and corals (Fig. 1) [1]. Research on cnidarians has contributed significantly to our understanding of biology over the last century. The concept of a developmental organizer, for example, was first discovered over 100 ago years by Ethel Browne using *Hydra* [2]. Green Fluorescent Protein (GFP), a tool used in molecular biology labs around the world, is derived from jellyfish, as are many other common fluorescent proteins [3]. Research on cnidarians has provided important knowledge on a multitude of biological processes, including regeneration, neurobiology, and development. In addition, the phylogenetic position of cnidarians as an early diverging animal lineage helps provide insight into the diversity and evolution of animal life. Given the prominent role cnidarians play in both benthic and pelagic realms, further insights into cnidarian biology, e.g., the effects of environmental changes on symbiosis and physiology, will also help us better understand and address the effects of climate change on ocean ecosystems.

**Fig. 2:**
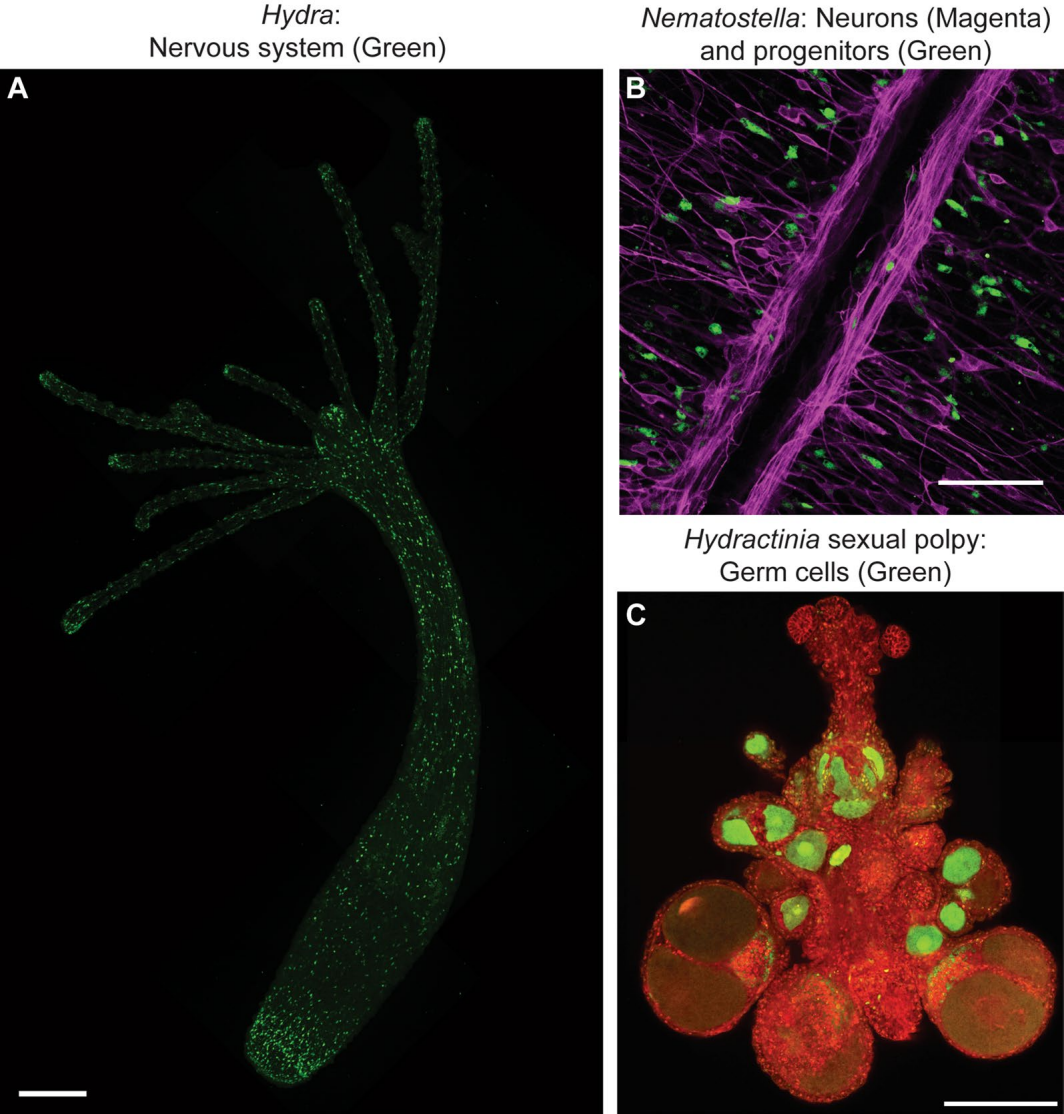
New technologies drive cnidarian research. **A** A whole *Hydra* polyp expressing GFP in all neurons imaged by confocal microscopy [46]. **B** Close up confocal section of the *Nematostella vectensis* gastrodermis from a line containing a CRISPR-Cas9 knock-in of GFP into the *Piwi1* locus (Green) and the *NvSoxB(2)::mOrange2* transgene [22, 47]. **C** A sexual polyp of transgenic *Hydractinia symbiolongicarpus* bearing the *β-tubulin::mScarlet* and *Piwi1::GFP* transgenes [48, 49]. All transgenic cells in the polyp are derived from a single transplanted stem cell (i-cell) [21]. Image credits: Ben Cox (**A**), Paula Miramon-Puertolas (**B**), Áine Varley (**C**). Scale bars: 250 µm (**A**), 50 µm (**B**), 200 (**C**)

Cnidofest 2022 (https://www.cnidofest.org/) was organized to bring together researchers working on cnidarians to share ideas and technical breakthroughs, and foster communication and collaboration in the community. The meeting was the second in this series after Cnidofest 2018 [4], which grew out of the first meeting Hydroidfest 2016 [5], and was a welcome return after a four-year hiatus due to Covid-19. The meeting, which was held at the University of California, Davis during a record-breaking heat wave, featured 45 oral presentations from selected abstracts. Approximately 50% of the talks featured the hydroid *Hydra vulgaris* and the sea anemone *Nematostella vectensis*, the two most commonly used cnidarian research organisms. This represents a reduction compared to the 2018 meeting, reflecting the continuous push of the community to work with a diversity of species to address new questions. In addition, we saw that technical innovations (e.g., gene editing) continue to expand the genomic and functional toolkit for all cnidarian research organisms (Fig. 2). Along with the selected talks, the meeting also featured three invited technology talks covering the topics of single-cell genomics (**Diego Calderon**, University of Washington) Cryo-EM (**James Letts**, UC, Davis), and in-vivo imaging (**Anupama Hemalatha**, Yale University) and a keynote by **Mansi Srivastava** (Harvard University) on the cellular and developmental biology of regeneration in acoels. Given the expansion of tools and organisms, the growing number of early career researchers, and the vibrant and supportive community present at this meeting, the cnidarian field continues to thrive and has a clear upward trajectory.

## Beyond a resource: cnidarian genomes provide insights into exciting biology

The meeting opened with a session on cnidarian genomics. Since, the first cnidarian genomes were published more than 15 years ago [6, 7], the field has seen a significant increase in the number and quality of sequenced genomes. The meeting highlighted many of these exciting recent advancements. **Jack Cazet** (Graduate student, Juliano lab, UC Davis) kicked off the meeting with a description of the most recent *Hydra* genomic resources from the Juliano Lab [8]. This included a high-quality draft genome for *Hydra oligactis* (produced in collaboration with the Hobmayer lab) and a chromosome level assembly of the *Hydra vulgaris* strain AEP genome, which is of particular interest as it is the strain used for transgenesis. Cazet also showed new data profiling cis-regulatory regions in *Hydra vulgaris*, as well as an updated *Hydra vulgaris* single-cell RNA sequencing (scRNAseq)-based cell atlas. Integrating the *Hydra* cell atlas with a recently published scRNAseq-based cell atlas from *Clytia hemisphaerica* [9], a hydrozoan with a life cycle including a medusa, allowed Cazet to identify putative conserved regulators of cell differentiation and opens new avenues to investigate cell type evolution.

Continuing with *Hydra*, **Tetsuo Kon** (University of Vienna) analyzed the genomes of the three cell lineages in *Hydra vulgaris* (the interstitial, ectodermal, and endodermal lineages) each of which is supported by an independent stem cell population [10]. Given the extreme longevity of *Hydra* and the absence of mixing between the lineages, Kon performed this analysis to uncover how the genomes of the three lineages may have evolved independently during asexual reproduction within the same organism. Kon analyzed transposon content within the lineage genomes as well as in genomic data from animals subjected to several rounds of regeneration from small fragments. Finally, by re-analyzing published RNA-seq data, Kon showed that many transposons have cell type-specific expression, thus opening the door to understand their putative functional relevance. **Sally Chang** (Postdoc, Baxevanis lab, NHGRI) presented the first draft genome assembly of *Podocoryna carnea*, a hydrozoan that possesses a medusa stage, unlike the closely related *Hydractinia* species which have lost this life cycle stage. Chang showed how comparisons to the *Hydractinia* genome can reveal the genomic consequences and/or causes of medusa loss.

Finishing up the session, **Karly Higgins-Poling** (Graduate student, Dawson lab, UC Merced) discussed symbiosis, a recurring theme throughout the meeting. Higgins-Poling used long-read sequencing to assemble the genome of *Mastigias papua*, a scyphozoan jellyfish native to the marine lakes of Palau that can exist either with or without endosymbionts. The genomic data are being used to assess the coevolution of Scyphozoa and their endosymbionts. Altogether, this session revealed how the collection and analysis of cnidarian genomic data are not just an important resource but is also being used to answer exciting biological questions.

## Cnidarians provide insights into the evolution of both novel and conserved processes

The phylogenetic position of cnidarians as sister group to bilaterians (Fig. 1A) makes them informative for providing evolutionary insights into the evolution of cnidarian and bilaterian traits. **Yareli Alvarez** (Graduate student, Babonis lab, Cornell University) discussed the sea anemone *Metridium senile* (Fig. 1B), which attacks unrelated individuals using fighting tentacles that contain specialized defensive stinging cells (cnidocytes). Cnidocytes are a broad class of cells that typify the cnidarian phylum. When needed, these fighting tentacles are generated through the transformation of feeding tentacles. Alvarez analyzed the process of tentacle transformation and showed a progressive loss of feeding cnidocytes and the concomitant gain of defensive cnidocytes from putative stem-like cells residing at the base of the tentacles.

**Anna Klompen** (Graduate student, Cartwright lab, University of Kansas) also presented work on cnidocytes, focusing on the toxin repertoire of *Hydractinia symbiolongicarpus* [11, 12]. *Hydractinia* is a colonial hydrozoan and displays a division of labor: colonies contain multiple polyp types with specific functions including predation and defense. Klompen generated a cnidocyte-specific, fluorescent transgenic reporter line and used this to show that different polyp types have a distinct cnidocyte repertoire. Using transcriptomes specific for both polyp types and for cnidocytes, she showed that several venom-like toxins are differentially utilized by cnidocytes in different polyp types. Finally, homologs of the well-studied and highly potent jellyfish toxin (JFT) family were identified, one of which is specifically expressed in a polyp type specialized for prey capture. **Alonso Delgado** (Graduate student, Daly lab, Ohio State University) continued the discussion on toxins by presenting the evolution of insulin-like peptide (ILP)-type toxins in anthozoans. Delgado showed that one group of these toxins shares sequence similarities to the highly toxic ILP toxins from cone snails, which appears to have evolved convergently.

Finally, **Patrick Steinmetz** (Michael Sars Centre, University of Bergen) presented recent work from his group on the evolution of nutrient uptake and transport during vitellogenesis in *Nematostella* [13]. Using fluorescent beads, labeled lipids and pulse-chase experiments, they demonstrated that nutrients are taken up and rapidly shuttled through the gonad epithelium into the extracellular matrix (‘mesoglea’) and into oocytes. This is likely carried out by conserved sets of lipid-transporting apolipoproteins and low density lipoprotein receptors expressed in the gonad epithelium and oocytes, respectively. Together, these data support the hypothesis that lipid transport via the ECM predates the last common ancestor of cnidarians and bilaterians. Altogether these talks highlighted that cnidarians are used to explore the evolution of novelty, but also to study deeply conserved mechanisms between cnidarians and bilaterians.

## Repeated losses and gains provide insights into life cycle evolution

A typical cnidarian life cycle consists of a free-swimming larval stage, a sessile polyp stage, and, in the medusozoan clade, a freely swimming medusa. In addition, most species undergo some form of asexual reproduction. Although asexual reproduction is well understood in a few species, most notable budding in *Hydra* which has been extensively studied, it is vastly understudied in most cnidarian groups. **Layla Al-Shaer** (Postdoc, Layden lab, Lehigh University) discussed the regulation of asexual reproduction in *Nematostella* which occurs through fission [14]. Using a newly developed quantitative approach to analyze fission, Al-Shaer showed that *Nematostella* has a set fission point and that the plane of fission is fixed along the oral-aboral axis of the polyp. Al-Shaer then demonstrated that fission rates increase with lower population density but could not find transcriptional differences between animals from low- and-high density populations. One possibility is that density-dependent repression of fission is mediated by a secreted chemical whose concentration increases with higher population density. Continuing with *Nematostella*, **Julia Baranyk** (Graduate student, Nakanishi lab, University of Arkansas) discussed the role of the cnidarian-specific neuropeptide RPamide in controlling life cycle timing in *Nematostella*. Baranyk showed that RPamide is dynamically expressed throughout the life cycle. The observation that RPamide-mutated animals progress faster through the larva-polyp transition suggests that RPamide acts as a negative regulator of this process.

Several oral presentations covered the evolution of cnidarian life cycles. **Lucas Leclère** (Sorbonne Université) presented work on the scyphozoan *Pelagia noctiluca* (Fig. 1D), which has lost the polyp stage and has an exclusively pelagic life cycle. Leclère presented new genome assemblies for *Pelagia* and the closely related, but polyp-bearing *Chrysaora colorata*, and discussed how these data can be used to study life history transitions. In addition, Leclère showed ATAC-seq and scRNAseq datasets across all stages of the *Pelagia* life cycle. Together, these resources make *Pelagia* an exciting new scyphozoan research organism to study life-cycle evolution and medusa development. **Matthew Travert** (Graduate student, Cartwright lab, University of Kansas) then discussed the repeated loss of the medusa stage throughout Hydrozoa. Loss, or reduction, of the medusa has occurred at least 15 times but the molecular basis of these losses is unknown. Using a comparative approach, he showed that the loss of the *tlx* gene, an otherwise well conserved homeobox gene, correlates with medusa loss. Notably, *tlx* is absent in studied anthozoans and endocnidozoans, and in those hydrozoans that completely lack a medusa stage. These observations led to the hypothesis that *tlx* gene loss is linked to medusa loss, which is further supported by *tlx* expression being restricted to the developing medusa in the hydrozoan *Podocoryne carnea* [15]*.* **Maciej Mańko** (University of Gdańsk) discussed the enigmatic life history of siphonophores, which form the most complex colonies among cnidarians (Fig. 1C). Their colonies are composed of zooids (i.e., specialized polyps) which perform different functions and are produced in a regulated manner to generate iterative clusters called cormidia. In some species these can detach and produce a sexually reproducing stage, the so-called eudoxoids. Little is known about this aspect of the siphonophore life cycle. Mańko showed that eudoxoid release evolved once in a monophyletic clade of siphonophores and that this was accompanied by the evolution of a new muscular structure required for their release. **Anush Kosakyan** (Czech Academy of Sciences) presented work on myxozoans (Endocnidozoa), a group of cnidarians that have undergone a radical life history change to become parasitic. Focusing on *Sphaerospora molnari*, a parasite of common carp, Kosakyan presented new transcriptomic and genomic data, which were used to identify genes associated with their parasitic life cycles. Finally, **Tsuyoshi Momose** (CNRS/Sorbonne Université) discussed the origin of the medusa body plan using *Clytia*, which is arguably the best-established cnidarian research organism with a medusa life cycle stage. Momose discussed the development and evolution of the entocodon, a tissue layer specific to developing hydrozoan medusae and derived from the endodermal layer. Momose suggested that a large scale heterochronic shift underlies the evolution of the medusa stage. Overall, the cnidarian phylum includes many life cycle shifts, which provides an excellent platform for revealing the molecular changes that drive such evolutionary transitions.

## Developmental and stem cell biology: new methods provide insights into classical questions

Cnidarians have been powerful research organisms in the study of development and stem cell biology for decades, a trend which continues and was exemplified by the large number of talks on this topic at Cnidofest 2022. Single-cell sequencing has become a powerful component of the developmental biology toolkit and is also used more and more in cnidarian research. The methodologies in this area are continuingly advancing and so, to provide an update on this area, we first moved away from cnidarians to hear from the first of three invited “technology speakers”. **Diego Calderon** (Postdoc, Trapnell Lab, University of Washington) demonstrated the power of applying single-cell genomics and computational approaches to understand development in *Drosophila melanogaster*. Calderon generated scRNA-seq and single-cell ATAC-seq datasets, consisting of over 1.5 million cells in total, spanning *Drosophila* embryonic development using overlapping 2-h time windows. Taking advantage of both the overlapping time windows and the slight asynchrony in development allowed for the generation of an atlas which represents a continuum during development rather than a set of discrete time points. Using these data, he reconstructed cellular lineage hierarchies with unprecedented resolution, and mapped the precise order of gene expression and regulatory events that underlie these processes. Using this resource, Calderon identified transcription factors with previously undescribed putative roles in mesoderm and neuroectoderm development. Overall, this approach marks a major technical advance and its application in other animals, including cnidarians, will undoubtedly reveal new and exciting biology [16].

Returning to cnidarians, the following talks addressed the roles of cell signaling in cnidarian development. **Fredrik Hugosson** (Postdoc, Martindale lab, Whitney Laboratory for Marine Bioscience, University of Florida) presented data tackling long-standing questions of the mechanistic basis of Wnt signaling activity in *Nematostella* and on the redundant roles of Wnt ligands and receptors in patterning the oral-aboral axis. Next*,* **Keith Sabin** (Postdoc, Gibson lab, Stowers institute) showed data on the development, cell type composition, and molecular patterning of the *Nematostella* apical organ. Using scRNA-seq, Sabin identified two cell types making up the apical organ: sensory cells and a ring of putative support cells. After confirming previous work that FGF signaling is required for apical organ development, Sabin went on to show that the same pathway specifies the two cell types of the apical organ in a dose-dependent manner. Finally, Sabin identified a homeodomain transcription factor (*prd146*) required for apical organ formation. *prd146* mutants lack apical organs but are competent to metamorphose, indicating that the apical organ is not required for this process. Given the lack of knowledge on the role of the apical organ in cnidarians, these animals represent an exciting new tool to dissect its function. **Kerstin Ohler** (Graduate student, Hassel lab, Philipps-Universität Marburg) continued the discussion of FGF signaling, presenting data in *Hydra* suggesting a role for FGF signaling in directing interstitial stem cell differentiation and in providing regional differentiation signals. **Masha Brooun** (Lunenfeld-Tanenbaum Research Institute) discussed the role of Hippo signaling in *Hydra*, showing that nuclear HyYap, the Hippo signaling effector, is graded along the body column and that Hippo signaling acts upstream of Wnt signaling as a positive regulator of budding. Broun additionally showed that knockdown of Lats, a negative regulator of Yap, affected epithelial morphology and led to both an absence of budding and shorter tentacles [17].

**Charisios Tsiairis** (Friedrich Miescher Institute for Biomedical Research) further discussed *Hydra* tentacle development by studying the role of the transcription factor *zic4* in tentacle formation and maintenance [18]. He showed that knockdown of *zic4* causes the transdifferentiation of battery cells, an epithelial cell type restricted to the tentacles, into basal disc cells, another epithelial cell type normally restricted to the foot. Finally, **Gaku Kumano** (Asamushi Research Center for Marine Biology, Tohoku University) presented the branched tentacles of the hydrozoan *Cladonema pacificum* as a model to understand the mechanisms and evolution of branching morphogenesis [19]. Kunamo showed that branching of the medusa tentacles occurs from the proximal end of growing tentacles. In addition, they showed that putative i-cells accumulating at the branching points contribute to multiple cell types in the new tentacle branches, and that FGF signaling is a likely regulator of branch elongation.

Cnidarian stem cells continue to fascinate biologists due to the extreme longevity, continuous cell renewal, and extreme regenerative abilities of these organisms. Much of our knowledge about cnidarian stem cells comes from decades of research in *Hydra*. The interstitial stem cells of *Hydra* are multipotent, giving rise to neurons, gland cells, nematocytes, and germ cells, but not to the epithelial lineages [10]. Stem cells in other cnidarians on the other hand have remained more elusive. Within the hydrozoans, previous studies in *Hydractinia* suggested that i-cells are pluripotent, giving rise to all adult cell types, which would suggest that i-cells differ in their developmental potential between hydrozoan species [20]. However, previous work had not tracked the fate of individual cells, thus leaving the potency of *Hydractinia* i-cells an open question. To test the potency of a single stem cell, **Áine Varley** (Graduate student, Frank lab, University of Galway) developed a method to transplant and follow single transgenically labelled i-cells in *Hydractinia* and confirmed that indeed, single i-cells give rise to all lineages and are, therefore, truly pluripotent [21] (Fig. 2C). In contrast to the well-studied i-cells of hydrozoans, the stem cell biology of anthozoans has remained a mystery. **Paula Miramón-Puértolas** (Graduate student, Steinmetz lab, Michael Sars Centre, University of Bergen) was able to take advantage of developments in CRISPR/Cas9 technology to endogenously label proteins in *Nematostella* as well as classical transgenesis tools to perform lineage tracing in juveniles and adults (Fig. 2B). She first showed that a population of stem-like cells located adjacent to the gonads gives rise to the germ cells. Intriguingly, however, these cells also produce neural-like cells and therefore represent a putative population of multipotent stem cells in juveniles and adults [22]. This exciting work lays a foundation for understanding stem cell biology in anthozoans, and for comparisons with stem cells in bilaterians and other cnidarians.

Together this broad session highlights the progress being made in understanding cnidarian development at the molecular and cellular level and illustrated the potential for new technologies to answer these questions.

## From formation to function of cnidarian nervous systems

The cnidarian nervous system shares a common origin with the bilaterian nervous system, making cnidarians a critical outgroup for understanding the evolution of nervous system development and function [23]. In addition, the relative simplicity of cnidarian nervous systems, which are diffuse nerve nets, makes them tractable systems to answer basic questions in neurobiology, such as how to build and regenerate a nervous system. Furthermore, cnidarians offer the opportunity to understand how a distributed nerve net can direct behaviors. However, we still have much to learn about the molecular details of nervous system development and function in cnidarians.

Two talks addressed the development of cnidarian nervous systems**.** First, **Abby Primack** (Graduate student, Juliano lab, UC Davis) took advantage of the continually self-renewing *Hydra* nervous system to build a neural scRNA-seq-based cell atlas that includes both differentiated neurons and cells in transitory states of neurogenesis. Primack used these data to build a differentiation trajectory of the entire nervous system and is using this resource as a starting point to dissect the gene regulatory network underlying the differentiation of the different neural cell types [24]. Next, **Minghe Cheng** (Graduate student, Layden lab, Lehigh University) focused on the role of *ashA* in *Nematostella* neurogenesis. *ashA* was previously shown to be required for the development of a subset of neural cells at the gastrula stage [25], but Cheng found that *ashA* also directs the development of a different set of *coup*-expressing neurons in the larval stage. These neurons do not require *ashA* function at the gastrula stage, indicating that *ashA* has a complex, stage-specific role in neural differentiation.

Moving from neurogenesis to nervous system function, **Wataru Yamamoto** (Postdoc, Yuste lab, Columbia University) and **Kelly Kim** (Graduate student, Robinson lab, Rice University) presented their work on *Hydra* neural circuit activity and behavior. *Hydra* has become a powerful model for understanding the neural control of behavior over the last number of years due to the creation of calcium indicator lines used to image activity of the entire nervous system in live, intact animals [26]. In addition, *Hydra* has a relatively simple repertoire of behaviors [27]. Yamamoto specifically discussed the somersaulting behavior of *Hydra*. Using live imaging, Yamamoto was able to break this complex behavior down into discrete steps and identify the patterns of neural activity associated with each step [28]. Kim then discussed *Hydra* phototaxis, which had been observed anecdotally but had never been systemically studied. She set up a controlled system to study phototaxis and observed that, although *Hydra* do indeed show a robust phototactic response, this only occurred in starved animals. She then used live imaging to address the precise steps in the phototactic response affected by feeding status. Future studies will determine the precise cellular and molecular basis by which the animals’ internal state controls behavior.

Overall, progress driven by new technologies is providing a more complete understanding of the cellular make-up of cnidarian nervous systems. In the future, combining this knowledge with the newly developed imaging and manipulation technologies illustrated here will provide mechanistic insights into nervous system function at the whole organism level.

## Looking under the hood: new insights into cnidarian cell biology

The recent development of new tools has led to an increased interest and ability to conduct studies at the subcellular level for a better understanding of evolutionary conserved processes and the discovery of new, fascinating cnidarian-specific biology. **Bert Hobmayer** (University of Innsbruck) showed new work on the Claudin family of proteins, which are major constituents of apical cell junctions in bilaterians. Hobmayer showed that the *claudin* gene family constitutes 33 orthologs in *Hydra* and then focused on Claudin-1, which is highly expressed in ectodermal stem cells and localizes to apical septate junctions. Knockdown of *claudin1* led to disruption of septate junctions, loss of epithelial integrity, loss of osmo-regulatory capacities, and to regeneration defects. **Marion Lechable** (Graduate student, Hobmayer lab, University of Innsbruck) discussed the Myc family of proteins, showing the independent diversification of the Myc family into 3 vertebrates and 4–6 cnidarian paralogs. This expanded cnidarian Myc family includes Myc3, a protein that lacks the N-terminal domain common to other Myc proteins. In *Hydra*, *myc3* is expressed in interstitial progenitors committed to neural and gland cell fate. To investigate the molecular function of Myc3, she demonstrated that the C-terminus of *Hydra* Myc3 fused to the N-terminus of v-Myc has high transforming capacity in an avian cell transformation assay. This demonstrated conservation of function of the C-terminus when fused to a functional N-terminus but the precise molecular function of this unusual Myc paralog in cellular differentiation remains a mystery [29]. **Bruno Gideon Bergheim** (Postdoc, Özbek lab, Center for Organismal Studies, Ruprecht-Karls-Universität Heidelberg) is using *Nematostella* as a model to investigate extracellular matrix (ECM) dynamics across development. To accomplish this, he generated proteomes from ECM at different life stages and showed a surprisingly rich and dynamic ECM composition.

The last two talks of this session focused on interesting aspects of *Hydractinia* biology. First, **Helen Horkan** (Graduate student, Frank lab, University of Galway) explored the resistance of this hydrozoan to DNA damage. She showed that a sub-lethal dose of irradiation caused an extended pause in proliferation, but that DNA repair occurs on a similar timeline to other organisms (~ 24 h). The role of this extended arrest is, therefore, unknown. She also unveiled a new scRNAseq-based cell atlas for *Hydractinia*, which will be a starting point to study the cell-type-specific dynamics of DNA repair. Next, **Manuel Michaca** (Graduate student, Nicotra lab, Starzl Transplantation Institute, University of Pittsburgh) discussed the basis of *Hydractinia* allorecognition, which is the ability of *Hydractinia* colonies to differentiate self from non-self. When two colonies come into contact, this process leads to the fusion of closely related colonies and conversely leads to a defensive battle between unrelated colonies. Although the molecular basis of allorecognition consists of highly polymorphic, cell surface proteins known as Alr1 and -2, their expression and action in vivo has remained enigmatic [30]. Michaca presented the development of new Alr1-specific antibodies, which revealed that Alr1 localized not only to cell membranes in stolons (where allorecognition occurs), but also is present in all tissues. This expression pattern supports the hypothesis that Alr1 mediates allorecognition, but also suggests that the allorecognition system evolved from pre-existing cell adhesion molecules. Overall, this session highlights that cnidarians have interesting cell biology which has not yet been well explored. However, given that cnidarians are highly amenable to imaging combined with new technological advances, we expect many more impactful discoveries in the future.

## The physiological response of cnidarians to environmental challenges

Since our last meeting four years ago, we have entered a structural biology revolution that is impacting many biological fields of study. Therefore, our session on physiology was kicked off by our second technology speaker, **James Letts (UC Davis)** who showcased the power of cryo-electron microscopy (cryo-EM) to study native protein complexes and to unravel new biology from understudied groups of organisms. Letts’s group focuses on mitochondrial electron transfer chain (mETC) complexes. He first showed work on the Mung bean (*Vigna radiata*) where they used Cryo-EM to obtain structures of mETC complexes, which in some cases were previously undescribed in any plant [31, 32]. Analysis of these structures revealed numerous differences in the structure, composition and the interaction between the complexes compared to opisthokonts (i.e., group including animals and fungi) and highlighted the variation in mETC complexes existing in nature. He then showed their work using Cryo-EM on mETC complexes isolated from *Tetrahymena*, a ciliate that is distantly related to both plants and opisthokonts. Impressively, instead of individually purifying complexes, the use of cryoEM allowed them to collect data from a single mixed sample and subsequently separated the structures using computational methods. This work revealed that the Tetrahymena complex IV is extremely large compared to any known homologous structures. Approximately half of the mass of the complex represents novel subunits that were previously unannotated in the genome, prompting Letts to point out that we are now using structural biology to inform genomics! This further highlighted the potential to make new discoveries by studying the structure of conserved complexes in a larger variety of species, which will likely soon include some cnidarians [33].

The session on cnidarian physiology covered a large range of topics from symbiosis to anti-viral immunity. **Samuel A. Bedgood** (Postdoc, Weis lab, Oregon State University) addressed the role of the crosstalk between endosymbionts and their hosts during development. Previous work demonstrated that *Aiptasia* containing symbionts developed faster than their aposymbiotic (i.e., without symbionts) counterparts [34]. Bedgood used symbionts which do not provide the necessary nutrition to their hosts (i.e., heterotrophic) to show that symbiont-host communication and not nutrition mediates the effects on developmental rates. **Maria Valadez Ingersoll** (Graduate student, Gilmore lab, Boston University) discussed her work on symbiosis, nutrition and immunity in cnidarians and showed that upon starvation, the sea anemone *Aiptasia* upregulated NfKB levels but this was accompanied by a decrease in the expression of antimicrobial and stress response genes.

Continuing the theme of nutritional regulation of development, **Kathrin Garschall** (Postdoc, Steinmetz lab, Michael Sars Centre, University of Bergen) discussed the connection between nutrition and growth in *Nematostella*. Garschall showed that *Nematostella* can survive extreme periods of starvation leading to massive reductions in body size. Even after prolonged starvation, however, animals could rapidly re-grow once feeding resumes. To characterize the molecular regulation of shrinkage and regrowth, Garschall used a combination of RNA-seq and imaging-based assays to show that proliferation is tightly linked to nutrition, and that apoptosis plays a role during late stages of starvation. This work positions *Nematostella* as an exciting new genetic model to study the interplay between growth and nutrition.

**Cory Berger** (Graduate student, Tarrant lab, Woods Hole Oceanographic Institution) discussed circadian clocks, which are dependent on external factors such as light and temperature. While these factors are usually studied independently in laboratory experiments, mis-aligning external factors can lead to a sensory conflict. To understand this, Berger exposed *Nematostella* to aligned and mis-aligned light and temperature cycles and performed both behavioral and RNA-seq experiments. Berger found that while the rhythmic expression of many genes either increased or decreased, the core clock genes maintained rhythmic expression showing that this transcriptional network is robust to external perturbation [35].

Several talks addressed cnidarian immunity from diverse perspectives. **Lys M. Isma** (Graduate student, Traylor-Knowles lab, University of Miami) discussed an emerging threat to coral reefs, a disease known as Stony Coral Tissue Loss Disease (SCTLD), documented in Florida and the Caribbean. The cause of the disease is unknown but different coral species are differentially susceptible. Isma proposed that differences in susceptibility are due to differences in immune capabilities among coral species. Phagocytosis assays were used to show that highly phagocytic cells are more prominent in coral species with low susceptibility to the disease as compared to those with high susceptibility. **Allyson DeMerlis** (Graduate student, Traylor-Knowles lab, University of Miami) analyzed wound healing responses in *Pocillopora damicornis*, a reef building coral. RNA-seq data were collected at early time points post injury, which allowed DeMerlis to identify distinct sets of genes activated at different time points during wound healing including a set of putative immune related genes. Finally, **Yehu Moran** (The Hebrew University of Jerusalem) presented recent work from his group on the molecular nature of anti-viral immunity in *Nematostella* [36, 37]. While anti-viral immunity in vertebrates relies on the interferon pathway, invertebrates such as *Drosophila* and *C. elegans* use the RNAi pathway. In both vertebrates and invertebrates, RIG-I-Like Receptors (RLRs) recognize dsRNA to activate the anti-viral response. Moran showed that RLRs in *Nematostella* similarly bind to dsRNA, and exogenous dsRNA can silence gene expression indicating that RNAi does occur. In addition, Moran showed that dsRNA promotes apoptosis in *Nematostella* cells. This work lays a strong foundation to uncover the molecular evolution of animal anti-viral responses. Overall, the work discussed in this session emphasizes the importance of understanding how cnidarians interact with their environments, which is vital for protecting these animals from the threats of climate change and other insults.

## Keynote: regeneration and development from the acoelomorph perspective

The lab of **Mansi Srivastava** (Harvard University), our keynote speaker, uses the acoel worm, *Hofstenia miamia*, to study regeneration. Srivastava pioneered *Hofstenia* as a model during her postdoc [38]. Srivastava is, however, no stranger to cnidarians, having begun her career working on non-bilaterian metazoans including *Nematostella* [6]. Srivastava discussed an evolutionary framework in which to study and compare regeneration between species at different levels, e.g., the comparison of cell types between organisms versus the comparison of molecular mechanisms. Using this framework, Srivastava showed two exciting findings from her group. In the first, they used genomic approaches to dissect early wound response in *Hofstenia* and have identified Egr as a crucial transcription factor upregulated upon injury. Egr was required for the expression of developmental genes like *wnt-3* and *sp5*. Interestingly, components of this gene network are important for regeneration in several other animals, including cnidarians, and may, therefore, represent a conserved module connecting the injury response to regeneration [39, 40].

Next, Srivastava addressed the biology of neoblasts, the stem cells which are responsible for the extreme regenerative capacity of *Hofstenia* [41]. Using scRNA-seq they determined that the neoblasts are a heterogenous pool of cells, many of which appear to be lineage-specialized progenitors. Using labelling approaches, they discovered that the neoblasts are derived from a specific pair of blastomeres in the embryo. To test that these embryonic blastomeres are responsible for the regenerative abilities of *Hofstenia*, they labelled these cells during embryonic development and followed them into the adult. This was accomplished using a transgenic line ubiquitously expressing the photoconvertible protein Kaede. Upon photoconversion of the neoblast-generating blastomeres, the derivatives of these cells could be visualized in the adult. These cells were morphologically similar to what has been described for neoblasts and scRNA-seq confirmed they exhibit the known molecular characteristics of neoblasts. They then amputated the worms and followed regeneration and found that the labeled cells were indeed the source of the regenerated tissue. This work shows the power of *Hofstenia* as a new genetic model organism to understand regeneration.

## New models and new mechanisms: insights into cnidarian regeneration

Continuing the theme of regeneration, our final technology speaker **Anupama Hemalatha** (Postdoc, Greco Lab, Yale University) discussed the use of live imaging to study skin regeneration in mice. Cnidarians are particularly amenable to live imaging due to their transparent nature and Hemalatha showed new and exciting avenues which could be explored in the future. The high regenerative capacity of the skin is driven by epidermal stem cells. Aberrant activity of these stem cells, however, can cause cancer. Hemalatha uses a mouse model whereby an activating mutation in the *beta-catenin* gene causes aberrant outgrowths. Within 2–3 months, these are corrected, and the mutant cells are eliminated from the skin. Using this model, they asked how wildtype epithelial cells recognize mutant cells to mediate their outcompetition. They focused on metabolism and used optical redox imaging to track the metabolic state of skin stem cells in the live mouse over time. They showed that *beta-catenin*-mutant cells have a rapid reduction in the redox ratio, prior to any observable morphological changes. The surrounding wildtype (WT) cells also have at first a reduced redox ratio that selectively recovers over time and, as a result, WT cells outcompete the mutant cells. Using a different model driven by a mutation in Hras, they showed that Hras mutant cells are not outcompeted by the WT cells. In this case, the reduction in redox ratio of mutant cells is transient and recovers, showing that “winner” cell types differ in both mutant models by their ability to recover redox over time. They went on to show that both mutations lead to an increase in glucose-flux through the TCA cycle and hence oxidation, in line with the redox drop in the mutant cells. Only the “winner” Hras mutant cells have upregulated pyruvate to lactate flux. The hypothesis that cancer cells rely primarily on glycolysis (so-called ‘Warburg effect’) has been suggested as a universal rule but this work shows that oncogenic mutants can also rely on upregulated glucose oxidation with a decoupling of lactate levels from upregulated TCA cycle that characterizes the “winner” mutation.

The power of cnidarians to regenerate have fascinated researchers for decades. *Hydra* has classically dominated work on cnidarian regeneration but, as two talks from the Juliano lab showed, there is still much to learn from this animal. **Ben Cox** (Postdoc, Juliano lab, UC Davis) presented *Hydra* as an exciting model to study the process of cell invasion. During homeostasis, interstitial stem cells located in the ectoderm must invade the ECM to populate the endodermal layer with new neurons and gland cells. Cox showed that during *Hydra* head regeneration, the ECM is actively degraded, which may allow interstitial progenitor cells to move freely from the ectoderm to the endoderm. Together this makes *Hydra* an exciting organism in which to study cell invasion.

Next, **Sergio Campos** (Postdoc, Juliano lab, UC Davis) discussed a comparative approach to better understand regeneration. Although *Hydra vulgaris* is capable of whole-body regeneration, the closely related *Hydra oligactis* displays a low rate of foot regeneration. Campos used RNA-seq to identify molecular factors that could explain the differences in foot regeneration ability. Previous work had shown that Wnt signaling is upregulated in *H. vulgaris* as part of the general response to injury, but Campos found that this does not occur in *H. oligactis*, suggesting a possible mechanism for the observed differences in regenerative potential.

Although cnidarian regenerative studies have traditionally focused on *Hydra*, the ability to regenerate is widespread among cnidarians. In recent years, the community has expanded into many other cnidarians to address regenerative biology questions. **Sosuke Fujita** (Postdoc, Nakajima lab, Tohoku University) discussed work on the cellular basis of tentacle regeneration in the *Cladonema pacificum* medusa [42]. The tentacles have a population of stem cells which reside at the proximal side. Fujita showed that during regeneration, proliferating cells accumulate and form a blastema. Labelling experiments suggested, however, that the resident tentacle stem cells are not the source of the blastema cells. Rather, it was proposed that a local conversion of cells to a stem-like fate is responsible for the regenerative response.

**Chiara Sinigaglia** (Sorbonne Université) discussed work elucidating the mechanisms of regeneration in the *Clytia* medusa, a relatively new model for regeneration [43]. Upon partial amputation, the remaining fragment of the medusa umbrella rapidly reforms a circular shape. Regeneration of missing structures does not appear to be regulated by a morphogen gradient in this system, but rather by the topology of the radial smooth muscles which are rearranged during wound healing. The onset of regeneration correlates with the formation of “hubs” composed of radial muscle fibers, which function as positional landmarks and are associated with activation of Wnt signaling. Subsequently, regeneration of the mouth structures involves both local cell proliferation and cell migration to the site of regeneration. Overall, this work established the *Clytia* medusa as a powerful tool to study the interplay between mechanical forces and molecular regulation of regeneration**.**

Finally, **Miguel Salinas-Saavedra** (Postdoc, Frank lab, University of Galway) closed the meeting presenting his work on cellular reprogramming in *Hydractinia* [44]. Salina-Saavedra demonstrated that an amputated *Hydractinia* head devoid of stem cells can de novo form new stem cells and eventually regenerate an entire colony. Salinas-Saavedra dissected this process on the cellular and molecular level and identified a population of senescent cells which are induced in the injured head. Using an impressive set of genetic experiments, including optogenetics, they showed that these cells are responsible for inducing these so-called “secondary i-cells”.

These talks highlighted the usefulness of cnidarians for understanding regeneration and demonstrated the variety of regenerative mechanisms and processes present across the phylum. In the future, understanding the basis for this diversity will more broadly contribute to our understanding of the evolution and molecular regulation of regeneration.

## Poster sessions

In addition to the oral presentations there were also 36 posters presented at the meeting. These also covered a large variety of topics and organisms reflecting the diversity of research in the community. The quality of the posters was very high, and 6 presenters were awarded poster prizes. The work of these prize-winning posters will be highlighted here. Zeeshan Banday (University of Chicago) presented their work using cnidocytes in *Clytia* as a model to study the role of Transmembrane Channel-like 1 and 2 (TMC-1/2) proteins in mechanotransduction. Alondra Escobar (Graduate student, Robinson lab, Rice University) unveiled their progress in generating new transgenic tools to visualize neural activity in *Hydra* using Genetically Encoded Voltage Indicators (GEVIs). Namrata Ahuja (Graduate student, Dunn lab, Yale University) showed work on the assembly of the genome of *Nanomia Bijuga*, a promising new siphonophore research organism. Yamaly Barragan (Graduate student, Rodriguez lab, The City University of New York) discussed their work on the geographical distributions of sea anemone, *Phymactis papillosa* which has three differently colored morphotypes in nature. Ndotimi Apulu (Graduate student, Nakanishi lab, University of Arkansas) presented their work on the role of Notch signaling in hair cell differentiation in *Nematostella*. Finally, Abby Primack (Graduate student, Juliano lab, UC Davis) presented their work on characterizing neurogenesis in *Hydra* using single-cell RNA-seq.

## Conclusion

Cnidofest 2022 was overall a fantastic success which highlighted the continuing development of the cnidarian research community in the US and internationally. The large number of excellent talks by young researchers shows the future is bright for the community and, along with the continued development of new tools and introduction of an ever-expanding array of research organisms, promises many more exciting findings in the coming years. For example, cnidarians have a high level of plasticity and regenerative abilities that we have only just begun to understand. In fact, some cnidarians may even to escape aging. Even the cnidarian nervous system is highly regenerative and thus may hold important secrets to inform regenerative medicine. Furthermore, cnidarians possess largely the same molecular tool kit as bilaterians and thus will continue to provide important information about the evolution of development. However, the unique aspects of cnidarian biology also make these animals exciting and a platform for exploring the evolution of novelty. Finally, cnidarians are an important part of the ecosystem and in particular we need a better understanding of coral biology to protect these animals from the effects of climate change. We expect that the tools and methodology being used to study cnidarians in general can also be employed in the fight to save our coral reefs. We look forward to gathering the community once again on August 14–17, 2024 in Bethlehem, PA at Lehigh University to discuss these and other new developments in cnidarian research.

## Data Availability

Not applicable.
